# Earlier Detection of Brain Tumor by Pre-Processing Based on Histogram Equalization with Neural Network

**DOI:** 10.3390/healthcare10071218

**Published:** 2022-06-29

**Authors:** M. Ramamoorthy, Shamimul Qamar, Ramachandran Manikandan, Noor Zaman Jhanjhi, Mehedi Masud, Mohammed A. AlZain

**Affiliations:** 1 Department of Artificial Intelligence and Machine Learning, Saveetha Institute of Medical and Technical Science, Saveetha School of Engineering, Chennai 600124, India; saimoorthy123@gmail.com; 2 Computer Science and Engineering, Faculty of Sciences & Managements, King Khalid University, Dhahran Al Janub, Abha 64351, Saudi Arabia; sqamar@kku.edu.sa; 3 School of Computing, SASTRA Deemed University, Thanjavur 613401, India; srmanimt75@gmail.com; 4 School of Computer Science (SCS), Taylor’s University, Subang Jaya 47500, Malaysia; 5 Department of Computer Science, College of Computers and Information Technology, Taif University, P.O. Box 11099, Taif 21944, Saudi Arabia; mmasud@tu.edu.sa; 6 Department of Information Technology, College of Computers and Information Technology, Taif University, P.O. Box 11099, Taif 21944, Saudi Arabia; m.alzain@tu.edu.sa

**Keywords:** brain tumor, ML (machine learning), identification of brain tumor, CAD (computer-aided diagnosis), (AHCN-LNQ) adaptive histogram contrast normalization with learning-based neural quantization

## Abstract

MRI is an influential diagnostic imaging technology specifically worn to detect pathological changes in tissues with organs early. It is also a non-invasive imaging method. Medical image segmentation is a complex and challenging process due to the intrinsic nature of images. The most consequential imaging analytical approach is MRI, which has been in use to detect abnormalities in tissues and human organs. The portrait was actualized for CAD (computer-assisted diagnosis) utilizing image processing techniques with deep learning, initially to perceive a brain tumor in a person with early signs of brain tumor. Using AHCN-LNQ (adaptive histogram contrast normalization with learning-based neural quantization), the first image is preprocessed. When compared to extant techniques, the simulation outcome shows that this proposed method achieves an accuracy of 93%, precision of 92%, and 94% of specificity.

## 1. Introduction

Cancer is still a dangerous illness with several subtypes, posing numerous obstacles in biomedical research. Images and image sequences make up about 80% of all corporate and public unstructured big data. Image fusion is involved in the integration of multi-source complementary information of the image and constructs a new portrait. The main functionality of image fusion is the formulation of distinct, reliable, and accurate figures rather than the source image. At present, image fusion exists practiced in a vast range of applications such as medical imaging, remote sensing, surveillance, and computer vision. Image fusion is performed at distinct levels based on three groups formulated such as feature level, pixel level, and decision level [1]. Among those methods, feature level and decision level are involved in the integration of image features in terms of feature descriptors and probabilistic variables. However, that variable does not offer detailed descriptions of various information sources. This leads to a loss of information infusion process.

In this context, pixel level performs fusing of pixel by pixel of the source image. Generally, the advantage of pixel-level fusion is that it can exist in the transform and spatial domain of the image. Image fusion model based on spatial domain performs investigation of spatial saliency and gradient of images, spatial frequency, dense scale-invariant features, quadtree structure, and so on. Through the incarnation of those variables, weights associated with regions or pixels are identified, and the source image is combined in the form of linear or non-linear fusion results. However, this spatialdomain feature does not offer information about the source image accurately, and fused image content is lost.

The transformation of pixel-level fusion consists of a set of algorithms such as multiscale analysis, pyramid transforms, DCT, DWT, SWT, CVT, CT, NSCT, and so on. This technique provides multiscale transformation based on the construction of basic mathematical functions concerning the applicability and role of images. Consider an example, DCT is performed based on the texture features of the image periodically but fails to identify the point of images; DWT provides details of an image but is not efficient based on consideration of various directions [2]. Programmed techniques are being picked over manual strategies. Through this, it is observed that the transformation technique is not adequate for the extraction and preservation of source image salient information; this, in turn, reduces the performance of image fusion. To overcome that limitation sparse representation-based method emerges with the utilization of the transformation domain. Several multiscale analysis techniques are evolved based on over complete project source image dictionary, with the involvement of image reconstruction through linear combination.

Generally, an image dictionary is trained with the utilization of high-quality images and describes the standard structure of the portrait. Due to limited traits of image pixels, the universal image feature dictionary is insufficient based on the representation of image content for irregular details and causes over-smooth fused results. The ability to explain the discovered highlights in many image sets on various extensions achieves the optimum simplicity in defining the clinical determination and the behavior itinerary [3]. By using MRI imaging, radiologists easily analyze the pathology of a patient’s mind that uses the strategy called CT (computed tomography). Image inspection is used not only for the underlying diagnosis but also for planning, assessing the treatment plan’s feasibility, and other aspects of clinical consideration. X-ray image preparation depicts the cerebrum’s life structures in various planes and reveals data about static structures, as well as data about fundamental tissue uprightness and smooth motion [4].

Paper organization is as follows: a review of literature is deliberated in Section 2. Section 3 designates the AHCN-LNQ method and its methodology. Section 4 offers results and discussion, and Section 5 provides the conclusion and future scope.

### Related Works

Brain disease recognition is the most upcoming and reliable field by using a procedure called medical imaging. To perceive the exploration of brain disease, various image processing methods are used. The automated segmentation method for brain tissue using the enhanced k-means technique is presented in [5]. With variation in shapes and size, brain tissue having tumor exist in various location. Manually detecting a brain tumor is not only time-consuming but is also associated with human mistakes, especially when they may rely on knowledge as well as an understanding of medical pathologists. By using automatic classification, cancerous cell identification is conducted. To classify concurrent MR images, an author in [6] proposes Vgg-16, ResNet-34, Alex Net, ResNet-50, and ResNet-18 into inflammatory disinfectors, cerebrovascular, degenerative, and neoplastic categories. In addition, compared their classification performance to that of the state-of-the-art structures, which are pre-trained prototypes. It achieves an accuracy of 95.23% ± 0.6 with the ResNet-50 prototype with five prototypes. By using brain MRI images, a large anomaly was detected. Evaluating the physical analysis output of MRI images is used for prototype results for clinicians. Researchers presented a technique for locating and identifying malignant cells in the brain in [7], which involves five steps: pre-processing, histogram clustering, morphological operations, image capture, and edge detection. Out of a total of 100 brain images, they used 50 for scheme improvement and 50 for scheme testing. For identification and localization of the tumor, cancerous cells in the brain are studied in the proposed method. However, it is impossible to detect brain tumors through identification by using this technique. For detection of brain tumors with more accuracy, MRI is used, which is presented in [8]. For health well-being, it is difficult to find the tumor at an initial stage. There is various research carried out on tumor identification with more accuracy. Hence to find brain tumor intensity with high distinctness, CNN was proposed.

Another study [9] obtained filtered pictures as part of the pre-processing processes in processing the malignant cell of the brain, which was also necessary inside the cancerous region. Filtering segmentation is used as a perfect image method for noise removal. For cancerous cell and brain tumor identification, Otsu and cuckoo methods are introduced in [10], which use MRI to determine tumor identification and formation. By using noise and error-free images, input images are processed to detect tumors with more accuracy. For better identification, a spatial filtering method was used for image smoothening. By using the Otsu method, the input image pixel value is increased. The cuckoo method finds segmented pixels with tumor and image values. The author of [11] suggested brain tumor identification approaches that employ k-means segmentation and various MRI brain images to evaluate brain tumors. In [12] is a proposed novel method for identifying tumors by using BWT and SVM techniques? In extracting non-brain tissue, this method achieves 95% of accuracy. In [13], the author proposed a clustering method for image segmenting and identification. For tumor identification, the contour method is used for segmentation. In [14], the author introduces a Gaussian mixture model for extracting features and brain tumor detection. Brain MRNet using a neural network was introduced in [15]. This design is based on attention modules, as well as a hyper column approach for acquiring residual networks. In pre-processing stage, Brain MRNet was used.

Limitations of the existing technique are as follows:

- High cost due to existing method inbuilt patterns is complex;
- Precision was still a challenge, and the dice score was usually about 90 or less;
- In every spatial data, flattening layer uses leads to feature maps loss;
- The existing technique took more time for convergence;
- With the increase in dimensionality, training time is increased and less time-consuming;
- While network designing, when voxels amount is more, itcauses computational complexity;
- Due to more data set population, over the fitting issue is attained.

## 2. Research Methodology

**Figure 1:**
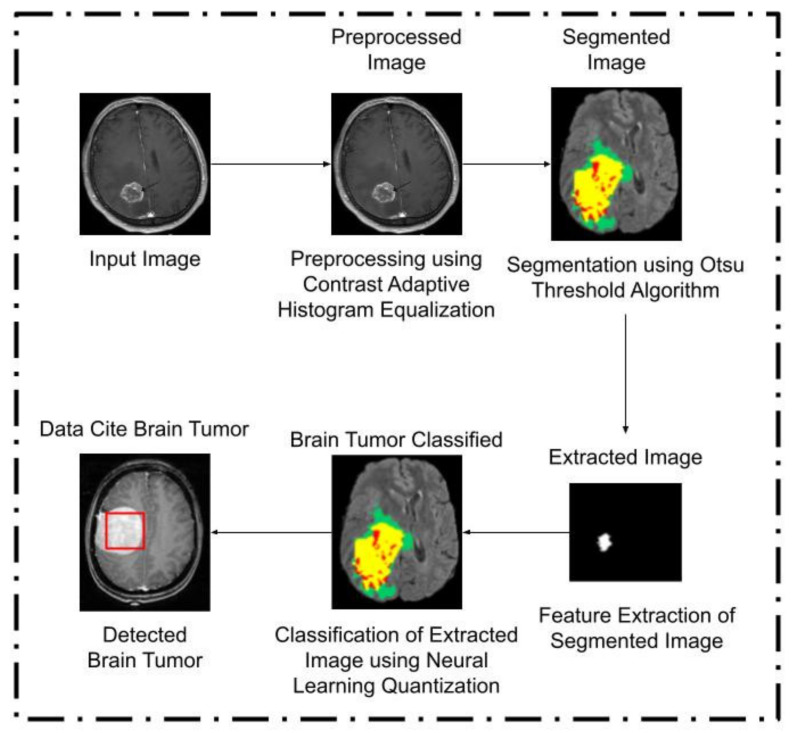
Proposed architecture of AHCN-LNQ.

For detecting brain tumors, a novel technique was introduced in this section. This method used an enhanced classification method with the pre-processing method. When compared with existing methods, this method achieves better performance. The proposed method architecture is as follows:

Figure 1 shows the architecture of the proposed method. For pre-processing input images and to remove noise, contrast adaptive histogram equalization is used. For segmenting images and to measure the image value threshold and extracted feature, the Otsu threshold is used. After extraction image is classified using neural learning quantization and brain tumor identification using CAD. Image enhancement has been achieved to acquire the precise uniform gray levels, which is translated by histogram for pixel mapping of gray level, by contrast, adaptive histogram equalization based on probability theory. For a novel image gray level value of a pixel is *r* (0 ≤ *r* ≤ 1) with a probability density is *p*(*r*), enhanced image with the gray level value ofa pixel is *s* (0 ≤ *s* ≤ 1) with probability density is *p*(*s*), and it is given by *s* = *T*(*r*). All equalized histogram bars have similar evolution by employing physics meaning of histogram, and it is expressed as (1). All of the formulas below refer to the Algorithm 1.

(1) $$\;p_{s}\left(s\right)ds=p_{r}\left(r\right)dr\;$$

*s* = *T*(*r*) has an increasing interval function and its inverse function is *r* = *T*^−1^ (*s*) is a monotonic process. Concerning Equation (1), Equation (2) is given as

(2) $$p_{s}\left(s\right)={\left[p_{r}\left(r\right)\frac{1}{ds/dr}\right]}_{r=T^{-1}\left(S\right)}=p_{r}\left(r\right)\frac{1}{p_{r}\left(r\right)}=1\;$$

For the convolution histogram equalization method, the mapping relationship is as follows:

For discrete states, correlation among *I* and *fi* is expressed in Equation (3)

(3) $$f_{i}=\left(m-1\right)T\left(r\right)=\left(m-1\right)\overset{i}{\underset{k=0}{\sum }}\frac{q_{k}}{Q}$$

where *m* denotes novel image gray intensities, *qk* is the image pixel with *k*th gray level, and the entire image pixel is *Q*. if an image with n gray intensities, *I*th gray intensity probability is *pi*, and its entropy is represented in Equation (4).

(4) $$e\left(i\right)=-p_{i}logp_{i}$$

Complete image entropy is given in Equation (5)

(5) $$E=\overset{n-1}{\underset{i=0}{\sum }}e\left(i\right)=-\overset{n-1}{\underset{i=0}{\sum }}p_{i}logp_{i}$$

With $p_{0}=p_{12}=\cdots =p_{n-1}=\frac{1}{n}$, *E* will attain its greatest. When histogram images are distributed uniformly, the entire image entropy attains its maximum level. It is more appropriate when equalization is enlarged by a dynamic range, which is represented in Equation (3). By using quantization of equalization, an interval is enlarged.

### 2.1. Pre-Processingusing Adaptive Histogram Contrast Normalization

For improved image, the popular and easy method used is called AHCN (adaptive histogram contrast normalization). In the output image, this method improves density distribution and its contrast. It mainly concentrates on dynamic image extending. To obtain maximum output, input image contrast is enhanced. So, the input image changes its visual quality and brightness. This method does not apply to unique intensity images. The image target is divided into tiles by using this histogram. DoF is attained by each tile in this technique. Using bilinear interpolation, boundary remapping density is executed to smooth tile. Using CDF (Cumulative Distribution Function), the histogram has limited amplification definite values. Above the histogram tile, clipped images are distributed into bins. In terms of image contrast, images are improved using AHCN. The pixel value of an original image is *a* $\left(0\leq a\leq 1\right),$ and its probability density is *p*(*a*), the pixel value of an improved image is *b* $\left(0\leq b\leq 1\right),$ and its probability density is *p*(*b*) and *b* = *T* (*a*) functions for mapping, and it is represented in Equation (6).

(6) $$p_{a}\left(a\right)da=p_{b}\left(b\right)db$$

For inverse function $b=T^{-1}\left(s\right)$ is represented in Equations (7) and (8) is

(7) paa=pbb1dadbb=T−1a

(8) $$p_{a}\left(a\right)=p_{\;b}\left(b\right)\frac{1}{p_{\;b}\left(b\right)}=1$$

The relationship between ‘*i*’ original image and ‘*f_i_*’ enhanced image is given in Equations (9) and (10) is

(9) $$f_{i}=\left(l-1\right)T\left(b\right)$$

(10) $$f_{i}=\left(l-1\right)\overset{i}{\underset{k=0}{\sum }}\frac{t_{k}}{Tn}$$

With *k*th intensity, $t_{k}$ is animage pixel in numerical. Considering an image with n intensity and probability of *i*th level is *pi*, then it is given in Equation (11),

(11) $$en\left(i\right)=-p_{i}\log p_{i}$$

For all images, the entropy is given as (12), (13),

(12) $$En=\overset{n-1}{\underset{i=0}{\sum }}en\left(i\right)$$

(13) $$En=\overset{n-1}{\underset{i=0}{\sum }}p_{i}\log p_{i}$$

where $p_{0}=p_{12}=\cdots =p_{n-1}=\frac{1}{n}$.

### 2.2. Segmentation Using OTSU Thresholding Method

If the segmentation is not performed correctly, the system’s subsequent phases will create misleading data, affecting the system’s overall performance. The suggested study uses OTSU’s techniques, which generate greater accuracy when detecting and are represented in Equation (14).

(14) $$\sigma^{2}=\frac{\sum_{i=0}^{N}{\left(X_{i}-\mu \right)}^{2}}{N}$$

where the image pixel value is denoted as $X_{i}$, mean is μ, and the number of pixels in one image is *N*. OSTU’s technique is utilized in a variety of image processing programs that perform histogram-based picture thresholding or convert grayscale images to binary. Images are divided into two intraclasses to calculate the optimum threshold. To investigate threshold values that reduce intraclass variance defined by two classes of weighted sum variances in Equation (15):

(15) $$\sigma_{\omega }^{2}\left(t\right)=\omega_{0}\left(t\right)\sigma_{0}^{2}\left(t\right)+\omega_{1}\left(t\right)\sigma_{1}^{2}\left(t\right)$$

where $\omega_{0}\;and\;\omega_{1}$ are two class weights probabilities that are separated by threshold *t*, $\sigma_{0}^{2}\;and\;\sigma_{1}^{2}$ are two classes of variance. As in Equations (16) and (17), OSTU evaluates that reduction in intraclass variance is equivalent to maximizing interclass variance.

(16) $$\sigma_{b}^{2}\left(t\right)=\sigma^{2}-\sigma_{\omega }^{2}\left(t\right)$$

(17) $$=\omega_{1}\left(t\right)\omega_{2}\left(t\right)\left[\mu_{1}\left(t\right)-\mu_{2}\left(t\right)\right]$$

where class probabilities are denoted by $\omega_{1}\;and\;\omega_{2}$ and class means is $\;\mu_{i}$.

Class probabilities $\omega_{1}\left(t\right)$ are evaluated from histogram t as in Equation (18):

(18) $$\omega_{1}\left(t\right)=\overset{t}{\underset{0}{\sum }}P\left(i\right)$$

${µ}_{1}\left(t\right)$ is class probabilities represented in Equation (19):

(19) $${µ}_{1}\left(t\right)=\overset{t}{\underset{0}{\sum }}P\left(i\right)\;x\left(i\right)$$

where *x*(*i*) is histogram b in center value.

### 2.3. Classification Using LNQ (Learning-Based Neural Quantization)

In general, NN has been utilized for architectural identification as well as perception in their results more than any other approach that has been documented in current works of ANN as an application. By utilizing neural theory, c (LNQ) is developed and enhanced predicated on learning quantization. To handle errors due to statistical measurement, LNQ shows neuron quantization number activation. The normalized triangular quantization statistics quantification was used in connection to the whole component in NL, as well as their vector input, with the most astronomically immense relationship rate equipollent to 1. Since LNQ is used to handle neural standards, their Euclidean distance has been transmuted in traditional LNQ by utilizing comparable neural values derived using the max–min operation between their input as well as vector reference. For accommodation of dual vectors in the network structure, the max–min operation has been altered. This network consists of one input, cluster, and output layer. The input layer is associated with neurons that are connected to hidden layer neuron clusters, and this is amalgamated with input data based on odor categorization. As a result, the odor category has a more immensely colossal number of neurons in each cluster for the hidden layer, where each cluster has neurons, and every neuron corresponds to one of the sensors. Every cluster has a neural codebook vector that is kenned by the category thatis represented. The input vector has been retrieved for each neural system, and each cluster does a homogeneous computation for learning quantization between vector input and their reference vector utilizing amended improved operation. To execute a minimum operation on each cluster, the propagated output is obtained in which it has enhanced similarity value from vector reference. When vector reference for learning quantization has been established adaptively in the cognition process, learning quantization may readily identify the vector input that relies on a statistical distribution of the input data. Quantization of separate standards is denoted by NL, and it is a weight or generation significance. For influence significance *w*, quantized version $\tilde{w}$ is given in Equation (20).

(20) $$\tilde{w}=w+n_{w}$$

where quantization noise is denoted by *n_w_*.

If *w* is uniform, Laplacian, Gamma distribution or Gaussian, SQNR, $\gamma_{w}$. The quantization procedure is given in Equation (21)

(21) $$10\log\gamma_{w}=10\log\frac{\epsilon \left(w^{2}\right)\;}{\epsilon \left(n_{w}^{2}\right)}\;\approx \kappa .\beta \;$$

where quantization efficiency is *κ* and *β* is the image bit-width quantizer.
**Algorithm 1**: **Procedure**: Using AHCN-LNQ, image features are learned and preprocessed.**Input**:$\;\alpha_{x}\;and\;\beta_{y}$**Output**: $\delta_{b}$1.In array image, calculating histogram of every contextual region about neighboring gray levels.2.Using CL value $N_{avg}=\frac{NrX\times NrY}{N_{gray}}$ to calculate contextual region contrast limited histogram. Where the average number of pixels is denoted as *N avg*, the number of gray levels is denoted as *N gray*, X and Y dimension number of pixels is denoted as *NrX* and *NrY.*3.$N_{CL}=N_{clip}\times N_{avg}$ is actual CL, then *clip N* has normalized CL with range [0, 1]. Pixels are clipped when *N CL* is less than the number of pixels.4.$N_{\sum clip}$ isatotal number of concise pixels, $N_{avggray}=N_{\sum clip}/$*N gray* is the gray level of standard pixels.5.Until the outstanding pixels are scattered, enduring pixels do reallocate.6.*P* (*i*) *input* probability of Clipped histogram, which is provided to create transfer process by enhancing intensity values.7.Within a sub-matrix contextual area, evaluating new gray level assignment of pixels.8.Find $\delta_{b}$9.End process.

## 3. Performance Analysis

Proposed architecture performance is explained in this section. Output image and its graphs are as follows:

### 3.1. Accuracy

It is several correct presages to the total number of presages. In terms of positives, precision is calculated for binary relegation, which is represented in Equation (22):

(22) $$\mathrm{Accuracy}\;=\frac{\mathrm{TP}\;+\;\mathrm{TN}}{\mathrm{TP}\;+\;\mathrm{TN}\;+\;\mathrm{FP}\;+\;\mathrm{FN}}$$

where: true positive is denoted as TP, false positive is denoted as FP, true negative is denoted as TN, and falsenegative is denoted as FN.

### 3.2. Precision

It is the ratio of true positive, and it is represented in Equation (23).

(23) $$Precision=\frac{\mathrm{TP}}{\mathrm{TP}\;+\;\mathrm{FP}}$$

### 3.3. Specificity

It is used to measure the actual negatives proportion, which is also called the true negative rate is represented in Equation (24).

Specificity = TN/(TN + FP).(24)

### 3.4. Image1–3

**Figure 2:**
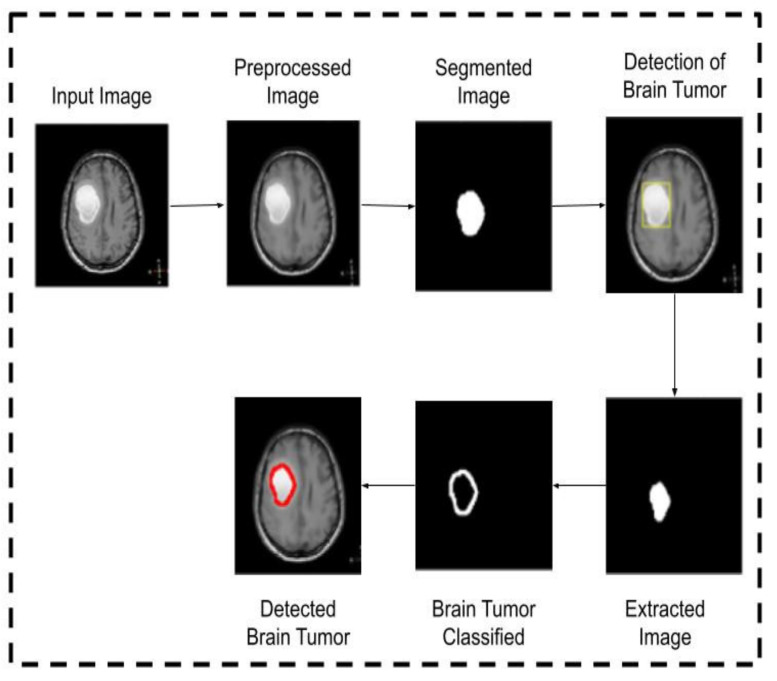
Operation of input image1.

**Figure 3:**
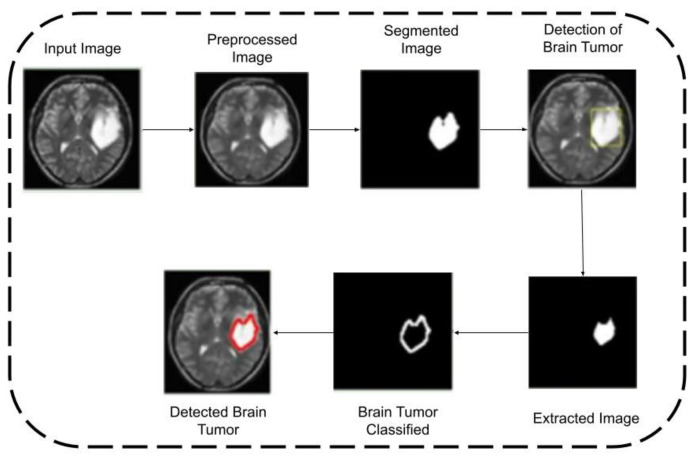
Operation of input image2.

**Figure 4:**
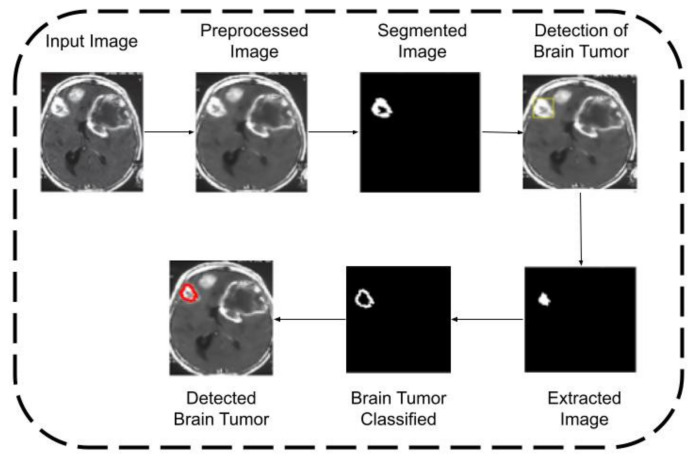
Operation of input image3.

Figure 2, Figure 3 and Figure 4 provide the architecture of images 1, 2, and 3. The first input image is preprocessed and then segmented. After that, a tumor is detected, which is then extracted using the feature extraction technique. A further brain tumor is relegated, and it is detected.

## 4. Results and Discussion

Table 1 represents a comparison of brain tumor detection by comparing NN, CNN, k-means, and proposed AHCN-LNQ.

**Figure 5:**
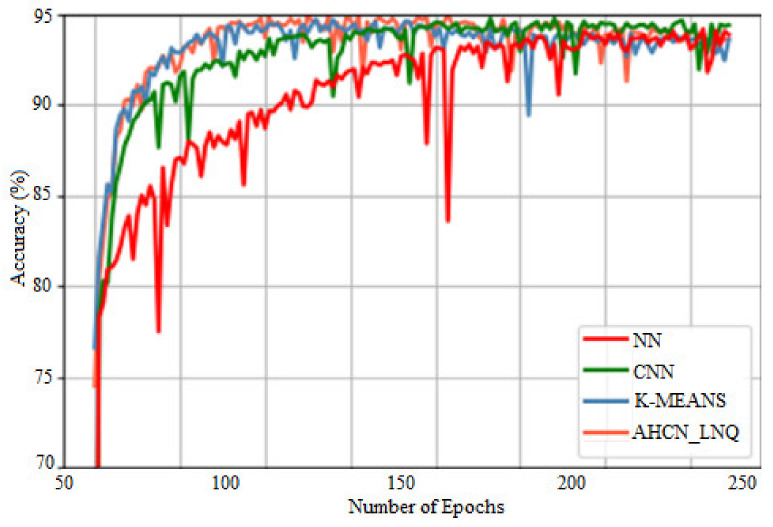
Comparison of accuracy.

Figure 5 shows a comparison of precision. The *X*-axis gives many epochs, and the *Y*-axis gives accuracy in %. When compared with subsisting methods such as NN, CNN, and K-MEANS, the proposed method attains higher accuracy for all images.

**Figure 6:**
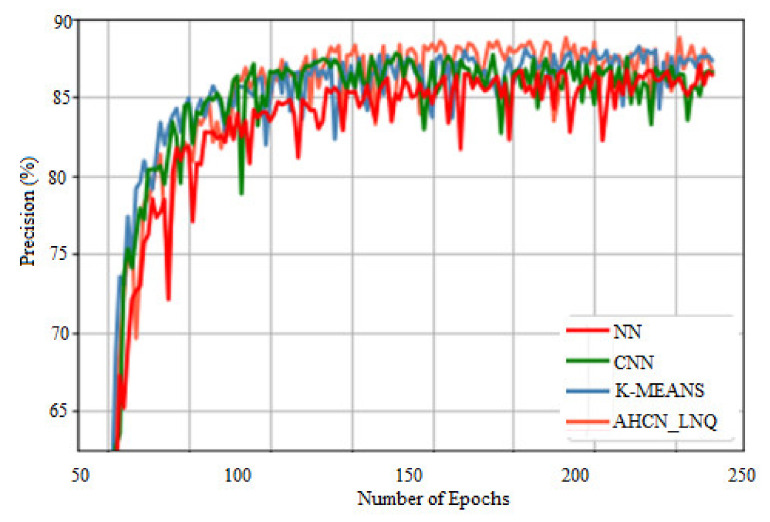
Comparison of precision.

Figure 6 represents a comparison of precision. The *X*-axis gives several epochs, and the *Y*-axis gives precision in %. When compared with other methods, the proposed method achieves higher precision for all images.

**Figure 7:**
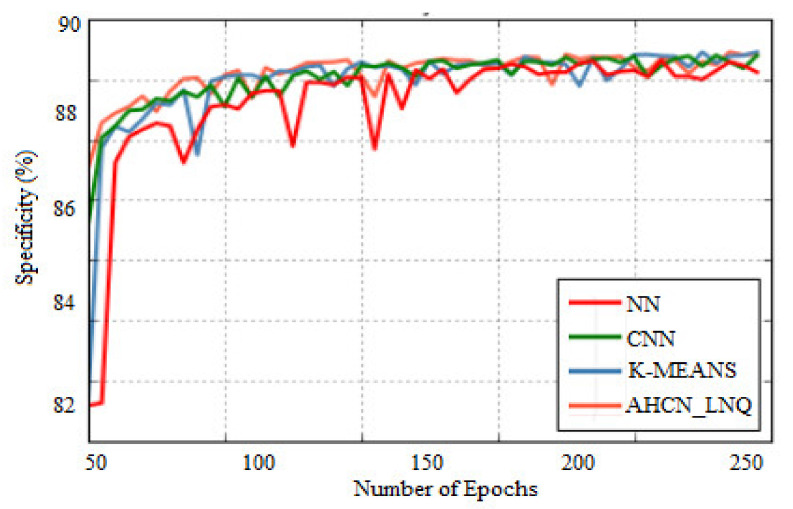
Comparison of specificity.

Figure 7 shows the comparison of specificity. The *X*-axis indicates some epochs, and the *Y*-axis indicates specificity in %. When compared with the existing method, the proposed method achieves higher specificity for all images. Table 2 shows a comparison of the recognition rate with the existing and proposed methods. When compared with the existing method, the proposed method shows a higher recognition rate.

**Figure 8:**
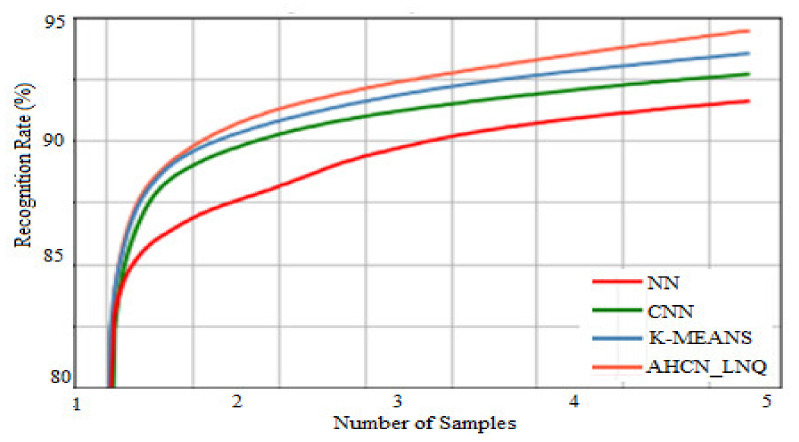
Accuracy of training sets.

Figure 8 gives training set accuracy. The *X*-axis gives thenumber of samples, and the *Y*-axis gives therecognition rate in percentage. When compared with existing techniques such as NN, CNN, and K-MEANS, the proposed method AHCN-LNQ achieves optimal accuracy.

### 4.1. Loss Function of Training Set

Table 3 represents a comparison of the testing set loss function. When compared with existing methods, the proposed method achieves a low loss function in terms of the training set.

**Figure 9:**
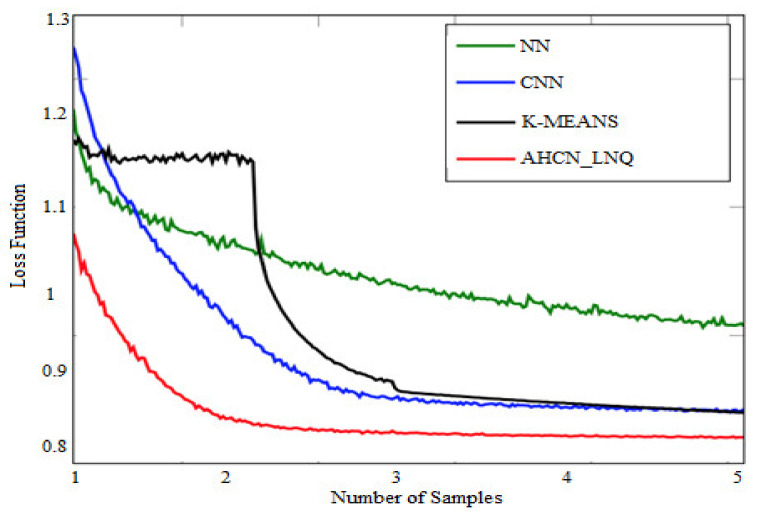
Loss function of training set.

Figure 9 shows the training set loss function. When compared with other existing neural network techniques, the proposed method achieves a lower loss function in terms of the training set in which the existing method achieves a higher loss function.

### 4.2. Loss Function of Testing Set

Table 4 gives the comparison of a testing set loss function. The proposed method achieves a low loss function in terms of the testing set when compared with existing methods.

**Figure 10:**
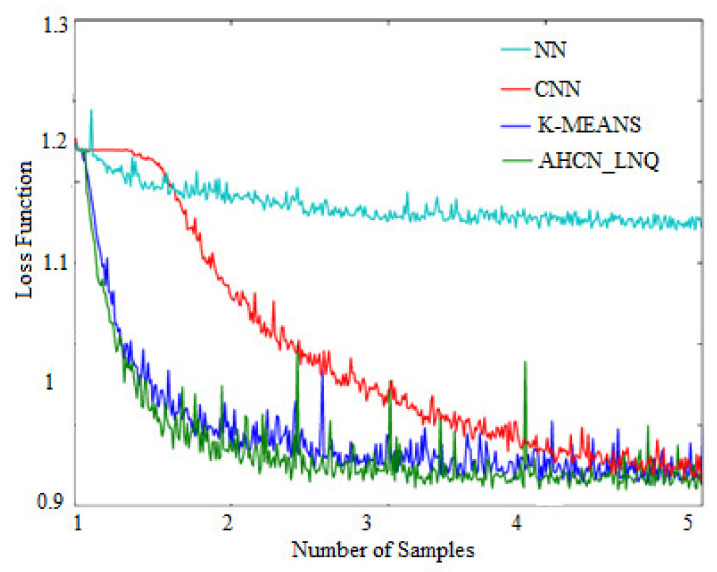
Loss function of testing set.

Figure 10 represents a comparison of the testing set loss function. When compared with other subsisting neural network techniques, the proposed method achieves a lower loss function in terms of the testing set in which the existing method achieves a higher loss function.

## 5. Conclusions

MRI is a consequential diagnostic imaging technology that exists utilized to detect pathological transmutations in tissues in addition to organs early. It is additionally a non-invasive imaging method. This paper proposes the detection of brain tumors using pre-processing through AHCN-LNQ. Detection and performance analysis of brain tumors conducted in an enhanced manner. In terms of precision, specificity, and accuracy, the proposed method achieves optimized outputs. Through proffered and subsisting method sample numbers, the apperception rate becomes premeditated. When compared with other neural network techniques, the proposed method accomplishes less loss function for both testing and training sets. When the number of samples increases, the loss function is maintained by the proposed technique. For all the parameters proposed technique obtained optimal output even for various input samples, and this method yielded optimized brain tumor detection in terms of accuracy of 93%, precision of 92%, and 94% of specificity. Future work includes detection of brain neural system disease and its symptoms earlier for various data sets.

## Figures and Tables

**Table 1:** Comparative analysis for brain tumor detection.

Parameters	NN	CNN	K-MEANS	AHCN_LNQ
**Accuracy**	92	93	94	95
**Precision**	85	86.5	89	89.5
**Specificity**	89	89.3	89.5	89.9

**Table 2:** Sample rate calculation on basis of recognition rate.

Samples	NN	CNN	K-Means	AHCN-LNQ
**1**	70	73	76	80
**2**	81	84	84	93
**3**	83	87	93	94
**4**	87	88	91	94
**5**	85	89	92	94

**Table 3:** Loss function for training set.

Loss Function of Training Set Samples	NN	CNN	K-Means	AHCN-LNQ
**1**	1.27	1.24	1.1	1.05
**2**	1.68	0.96	0.84	0.82
**3**	1.68	0.96	0.83	0.82
**4**	1.68	0.97	0.85	0.81
**5**	1.69	0.97	0.84	0.82

**Table 4:** Loss function for testing set.

Loss Function of Testing Set Samples	NN	CNN	K-Means	AHCN-LNQ
**1**	1.27	1.2	0.98	0.95
**2**	0.95	0.98	0.94	0.92
**3**	0.95	0.98	0.94	0.92
**4**	0.95	0.98	0.95	0.93
**5**	0.96	0.97	0.95	0.91

## Data Availability

Data will be shared for review based on the editorial reviewer’s request.
